# Retinal damages in turner workers of a factory exposed to intraocular foreign bodies

**DOI:** 10.4103/0019-5278.44696

**Published:** 2008-12

**Authors:** S. Masoud Shushtarian, M.S. Mirdehghan, P. Valiollahi

**Affiliations:** Tehran Medical Branch, Islamic Azad University, Tehran, Iran

**Keywords:** Electroretinogram, intraocular foreign body, retinal damages, turner workers

## Abstract

Damages caused by an intraocular foreign body (IOFB) to the visual system, mainly the retina, mostly occur during certain occupational activities. Turners are among the laborers who are mostly exposed to IOFB. The aim of the present work is to survey the effect of an IOFB on the visual system, mainly the retina. Fifty laborers of a turner factory who were exposed to IOFB were selected. Electroretinography (ERG) was recorded in all the laborers. Beside these workers, 50 laborers with no incidence of IOFB were also selected. They were also tested using ERG. The results obtained in the two groups were compared together to search for the possible changes in the two groups. The ERG patterns of the case groups were found to be changed in comparison to the control group. The changes were observed in the area under the b-wave of the ERG pattern in the early stage of damage and in the late stages, the latency and amplitude of the ERG b-wave were also affected. Finally, from the result of the present study, one can conclude that ERG is a suitable technique to search for the retinal changes in the laborers exposed to IOFB.

## INTRODUCTION

Certain hazardous factors in the working environment can damage the workers in different fields. Some of these factors are inevitable. For instance, the humidity in some occupational activities is unavoidable and this physical factor is reported to produce mental stress in workers of a cheese-processing factory.[1] On the other hand, most of the hazardous agents can be controlled to protect the workers in different factories. Intraocular foreign bodies (IOFB) produced by hammering or chiseling can damage the visual system of the workers in related fields. These tiny metal particles usually damage the retina due to their penetration to different retinal layers. There exist a large number of reference bases on the effect of these tiny particles jumping to different parts of the visual system.

Resch *et al*. worked on the damages produced by the incidence of the tiny metal particles on the cornea. They also tried to look for a confocal microscopic of increased Langerhans cell activity after corneal metal foreign body removal.[2]

There are quite a large number of reports regarding the cataracts produced by the penetration of these tiny particles into the lens in the visual system.[3–5]

The retina in the visual system can be damaged if the tiny metal particles go deep into the different layers of the retina.[6–7]

There are different techniques to look for the effect of the IOFB on the retina. Color photography, fluorescein angiography, optical coherence tomography and echography are among these techniques.[8]

Electrophysiological techniques are useful in this respect too.[9] The importance of electrophysiological techniques are reported for different pathological and non-pathological conditions.[10] Electroretinography (ERG) is among the techniques that may be used to survey the effect of IOFB on the retina because ERG can detect biochemical and functional abnormalities of the retina before a pathological change can be seen by ophthalmology or fluorescein angiography.[11–12]

The present work deals with the retinal damages observed in the laborers of a turner factory who were exposed to IOFB during the last 5 years.

## MATERIALS AND METHODS

In the present research work, 100 subjects, i.e. 50 normal, as a control group and 50 laborers exposed to IOFB as a case group were selected. The medical records of the control group show normal eye examination of the said group and the eye check up of the case group was also normal before the incidence of IOFB. The two groups selected were of the same age range. The laborer with absorption of foreign particles in the cornea and the lens were discarded from the case group, i.e. the case group was selected only from the population with penetration of foreign bodies into the retina.

ERG, which is a technique to record the electrical activity of the retina by applying light flashes to the visual system was recorded in the total population. Pantops – Pc_2_ was used for this purpose. For recording ERG, the subjects’ eyes were dilated using medriasil drops. After eye dilation, three electrodes were used to connect the subjects to the machine to record the ERG. The active electrode, which is a hepatic (scleral) contact lens, was fixed on the sclera.

The second electrode, i.e. the reference, which is a chloride silver earring was fixed to the ear lobe by conducting jelly. The conducting jelly was applied for better impedance matching. Finally, the earth electrode, i.e. the third electrode, which is a chloride silver plate was attached to the patient forehead.

Twenty-five flashes of light were applied to the subjects’ eye and the electroretinogram pattern was recorded in case of the total population. The ERG pattern consists of two clinically important negative and positive peaks, i.e. a- and b-waves, respectively.

In the present work, latency (msec) and amplitude (μV) of the b-wave of ERG were recorded. In fact, as we could not find a scientifically reasonable relation between the a-wave and IOFB, we omitted the a-wave measurement. The mean and standard deviations were calculated in case of the different groups. Finally, the SPSS program was taken into consideration to search for a difference in the results obtained in the two groups.

## RESULTS

IOFB in certain occupations may bring about retinal degeneration. The result of the present work is an evidence for this comment.

Table 1 is the mean amplitude/± SD and mean latency/±SD of the ERG b-peak in the control and case groups. The values are 115 ± 27 and 105 ± 35 μv for the amplitude of control and case group, respectively. The values for mean latencies are 42/2 and 51/8 msec for the control and case groups, respectively. As observed in the table, the P values for the two measurements are 0.058 and 0.000, respectively.

**Table 1:** Mean amplitude/±SD and latency/±SD of ERG b-wave in the control and case groups

	Amplitude	latency
		
ERG b-wave groups	± S.D	± S.D
	115	42
Control	——	——
	27	2
	105	51
Case	——	——
	35	8
	*P* = 0.058	*P* = 0.00

**Graph 1:**
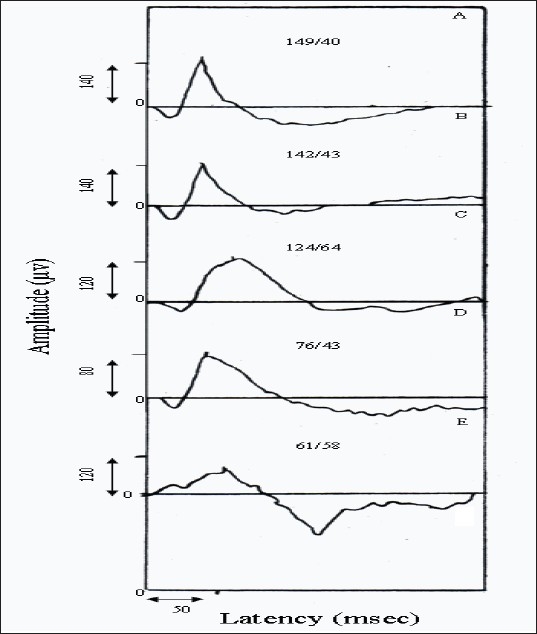
Sample ERG patterns with amplitude/latency measurement of the ERG b-wave in the following cases

Graph 1 is the sample ERG pattern in case of normal subjects (visual system) and four laborers who were exposed to IOFB.

The selection of samples is according to different patterns obtained for different groups of the laborers.

A: Normal laborer (normal visual system).

B: A laborer with very early retinal damages.

C: A laborer with deep retinal damages.

D: A laborer with deep retinal damage but normal amplitude and latency of ERG b-wave.

E: A laborer with severe deep retinal damages.

## DISCUSSION

The aim of the present work is an indication of the retinal damages produced by IOFB. ERG is a technique that reflects these damages in early stages, and the present discussion proves the advantages of this technique.

According to Table 1, there is a significant latency difference between the control and the case group and, on the other hand, the difference in amplitude is not significant in the two groups. The reason may be given by the fact that it is the latency of the ERG b-wave that increases in the early stages of deep retinal changes[12] and, in the latter stages of damages, there will be changes of amplitude or, in other words, reduction of amplitude of the ERG b-wave. In the present study also the laborers are taken for ERG measurements within the first days of exposure so that the changes are observed in latency rather than in amplitude.

Considering Graph 1 will also reflect a better sight to ERG changes due to retinal damages. Graph 1A is a sample ERG pattern in case of a normal laborer, i.e. a healthy retina should resemble an approximately similar type of ERG pattern.

Graph 1B is a sample ERG pattern of a laborer who is exposed to IOFB. Twenty-six percent of the exposed laborers showed approximately this type of pattern. Graph 1B is very similar to Graph 1A and the only difference is the slight increase in the area under the b-wave, which is an indication of very early changes of retinal function.[12–13]

Graph 1C is a sample ERG of a laborer with deep retinal degeneration. Forty-seven percent of the cases showed this type of ERG pattern. In this figure, the amplitude of the b-wave is within the normal range and there is an increase in the latency of the ERG b-wave. In fact, this type of ERG pattern is mostly observed in the laborers of the case group. Therefore, as explained previously, as far as IOFB exposure is concerned, it is mostly the latency that changes rather than the amplitude of the b-wave.

Graph 1D: this type of pattern is observed in 20% of the cases of laborers. In this pattern, both amplitude and latency of the ERG b-wave are within the normal range and the only difference is the area under the b-wave, which is obviously much more than the area of the ERG pattern in Graph 1A. In fact, in such a condition, the area should be taken into consideration otherwise the pattern is normal as far as amplitude and latency of ERG b-wave are concerned. Finally, in such type of a pattern, latency measurement from the tip of the peak may misguide the concerned ophthalmologist.

Finally, Graph 1E is a sample ERG pattern, which is observed in only 7% of the cases. It is a severe case of IOFB exposure, which produces deep retinal layer degeneration of the bipolar and muller cells. This type of degeneration decreases the amplitude of the ERG b-wave and therefore the damage of the two said layers. Furthermore, the degeneration also increases the latency of the ERG b-wave. This type of patterns leads the ophthalmologist to diagnose the degenerative effect of IOFB on the retina.

## CONCLUSIONS

The following are the conclusions of the present study:

1. IOFB, which is an important occupational hazard, may damage the retina and its functions.
2. ERG, which is a measure of the function of the retinal layer, is a suitable technique to survey the damages caused by IOFB to the retinal layers.
3. It is the latency of the ERG b-wave that increases due to early retinal damages following exposure to IOFB.
4. Severe damages of retinal layers due to IOFB reduce the amplitude and increase the latency of the ERG b-wave, respectively.
5. Finally, if the two parameters of the ERG b-wave, i.e. amplitude and latency, are normal, the area under the b-wave should be taken into consideration for proper decision regarding the status of the retinal layers.
